# Enhanced Surveillance for Meningococcal Disease—United States, 2015–2019

**DOI:** 10.1371/journal.pone.0319940

**Published:** 2025-06-09

**Authors:** Amy B. Rubis, Daya Marasini, Rebecca Howie, Basanta Wagle, Shalabh Sharma, Brittney Thomas, Sara Eisen, Sandip Shrestha, Henju Marjuki, Lucy A. McNamara

**Affiliations:** 1 Meningitis and Vaccine Preventable Diseases Branch, Division of Bacterial Diseases, National Center for Immunization and Respiratory Diseases, Centers for Disease Control and Prevention, Atlanta, Georgia, United States of America; 2 Applied Science Research and Technology, Contractor assigned to Meningitis and Vaccine Preventable Diseases Branch, Division of Bacterial Diseases, National Center for Immunization and Respiratory Diseases, Centers for Disease Control and Prevention, Atlanta, Georgia, United States of America; 3 Meningitis and Vaccine Preventable Diseases Branch, Division of Bacterial Diseases, National Center for Immunization and Respiratory Diseases, Centers for Disease Control and Prevention, Atlanta, Georgia, United States of America (now with U.S. Naval Medical Research Unit (NAMRU) SOUTH); McGill University, CANADA

## Abstract

**Background:**

High quality surveillance data are important to monitor U.S. epidemiology of meningococcal disease. Enhanced Meningococcal Disease Surveillance (EMDS) was established in 2015 to collect additional data and isolates from reported cases.

**Methods:**

Epidemiologic information and isolates obtained through EMDS for meningococcal disease cases reported through the National Notifiable Diseases Surveillance System (NNDSS) during 2015**–**2019 were included in the analysis. Isolates were characterized by serogrouping and molecular typing using either Sanger sequencing or whole genome sequencing. Case fatality ratios (CFRs) were calculated using cases with known outcome as the denominator. Odds ratios (ORs) were calculated using logistic regression.

**Results:**

A total of 1,806 meningococcal disease cases were reported during 2015**–**2019. The average annual incidence was 0.11 cases per 100,000 population. For key variables already collected through NNDSS, EMDS improved completeness over NNDSS by 21%-39% each year during 2015–2019. Completeness of key variables in EMDS improved over time by an average of 18%. Testing of isolates submitted through EMDS showed that serogroups B and C were predominant in all five years. For serogroup B, the most common clonal complex (CC) was CC32 in 2015**–**2017 and CC41/44 in 2018**–**2019; for serogroup C, CC11 was most common in 2015**–**2017 and CC103 in 2018**–**2019. The CFR was significantly lower for CC103 (6.7%) compared to other CCs (13.6%) (OR 0.44, 95% CI: 0.22–0.89).

**Conclusions:**

U.S. incidence of meningococcal disease remained low during 2015**–**2019. Data and isolates obtained from EMDS supplement NNDSS and are critical to monitor U.S. meningococcal disease epidemiology.

## Background

Meningococcal disease, caused by the bacterium *Neisseria meningitidis*, is a rare but serious illness [1,2]. Despite low overall incidence in the United States, several outbreaks have been reported in recent years among college students, people experiencing homelessness, and men who have sex with men (MSM) [3–6]. High quality surveillance data are important to monitor epidemiology in the U.S., understand risk factors for disease, and inform meningococcal vaccine policy.

Quadrivalent meningococcal conjugate (MenACWY) vaccines have been licensed and recommended by the Advisory Committee on Immunization Practices (ACIP) for routine use in persons aged 11–12 years since 2005, and a booster dose was recommended in 2010 for adolescents 16 years of age [7]. ACIP also recommends MenACWY vaccine for persons at increased risk of meningococcal disease, including a primary series of two or more doses, depending on age, and booster doses every 3–5 years for persons at increased risk because of certain underlying medical conditions (e.g., complement deficiency, asplenia, HIV infection).

In 2015, ACIP recommended that individuals aged 16–23 years may be vaccinated with a serogroup B meningococcal (MenB) vaccine based on shared clinical decision-making [3]. ACIP also recommends that persons aged ≥10 years at increased risk for serogroup B meningococcal disease because of certain underlying medical conditions (e.g., complement deficiency, asplenia) or increased exposure to *N. meningitidis* (e.g., microbiologists, populations at risk during outbreaks) receive MenB vaccine.

Meningococcal disease has been nationally reportable in the U.S. since 1920 through the National Notifiable Diseases Surveillance System (NNDSS). In addition, active surveillance has been conducted in 10 sites through Active Bacterial Core surveillance (ABCs) since 1996 as part of CDC’s Emerging Infections Program [8]. However, information collected through NNDSS is limited, and with the declining incidence of meningococcal disease, ABCs only captures a small number of cases each year. To address these challenges and gather more complete information on populations at increased risk for meningococcal disease, Enhanced Meningococcal Disease Surveillance (EMDS) was established in 2015 to collect additional data and isolates from reported cases. This report summarizes findings from the first five years of EMDS in the U.S. and highlights how the improvements to surveillance through EMDS have generated valuable information on the epidemiology of meningococcal disease during this period.

## Methods

Confirmed and probable meningococcal disease cases reported through NNDSS from 2015–2019 were included in the analysis. Cases were classified as confirmed or probable according to the Council of State and Territorial Epidemiologists surveillance case definition [9]. Confirmed cases were defined as isolation of *N. meningitidis* from a normally sterile body site (e.g., blood, cerebrospinal fluid [CSF]) or from purpuric lesions, or detection of *N. meningitidis*-specific nucleic acid in a specimen obtained from a normally sterile body site using a validated polymerase chain reaction (PCR) assay. A probable case was defined as detection of *N. meningitidis* capsular polysaccharide antigen in formalin-fixed tissue by immunohistochemistry or in CSF by latex agglutination.

Limited epidemiologic data are collected through NNDSS, including basic demographics, serogroup, and outcome, but a high proportion of data are missing or unknown [1]. As part of EMDS, from 2015, epidemiologic data and isolates were collected for cases reported through NNDSS from 44 state and 3 large jurisdiction health departments (95% of the U.S. population), and in 2019, this expanded to all 50 states and 3 large jurisdiction health departments. Key variables collected through NNDSS, including serogroup and outcome, were collected again through EMDS to improve completeness. The additional epidemiologic data collected included the following for 2015–2019: whether the case was part of a meningococcal disease outbreak according to CDC outbreak definitions [10,11]; if the case was in a college student, man who has sex with men (MSM), or person with HIV; and MenACWY vaccination history. Beginning in 2016, three additional variables were added: if the case was in a person experiencing homelessness (PEH), MenB vaccination history, and if the patient was taking a complement inhibitor (eculizumab or ravulizumab). All supplemental data collected through EMDS were linked to data submitted through NNDSS. Beginning in 2017, before data were finalized for each year, jurisdictions received feedback on missing data for the epidemiologic variables to request any additional information available to improve data completeness. Race and ethnicity were collected through NNDSS, but unlike serogroup and outcome, were not collected again through EMDS. To improve completeness of race and ethnicity, these variables were included in the feedback on missing data starting in 2017.

County of residence of patients was classified into one of six urban-rural categories according to the 2013 National Center for Health Statistics Urban-Rural Classification Scheme [12]. To further classify counties into urban versus rural, four of these six categories (large central metropolitan, large fringe metropolitan, medium metropolitan, and small metropolitan) were combined into an urban category, and two (micropolitan and noncore) were combined into a rural category.

### Laboratory methods

Serogrouping of *N. meningitidis* isolates was performed at state public health laboratories and all available isolates from associated cases were submitted to CDC as part of EMDS. Isolates were further characterized at CDC’s Bacterial Meningitis Laboratory by serogrouping and molecular typing to determine clonal complex (CC) using Sanger sequencing as described previously in the WHO manual [13] or whole genome sequencing (WGS) using Next-Generation Sequencing (NGS) technology, depending on year. WGS was conducted by Illumina platform (Illumina Inc., San Diego, CA, USA) using sequencing libraries prepared with NEBNEXT Ultra DNA library preparation kit (New England Biolabs, Ipswich, MA, USA). Pair-end illumina sequencing data was analyzed and characterized using Bacterial Meningitis Genome Analysis Platform [14]. If serogrouping information was unavailable from the state or if WGS contradicted the state result, slide agglutination was additionally conducted at CDC’s Bacterial Meningitis Laboratory for serogroups A, B, C, W, X, Y, Z, and Z’/E [13]. If an isolate was not available for submission to CDC, the state laboratory serogroup result was used.

### Statistical analyses

Data were analyzed using SAS version 9.4. Differences in completeness proportions for NNDSS compared to EMDS were tested using McNemar’s exact test. Incidence rates were calculated using National Center for Health Statistics’ (NCHS) bridged-race postcensal population estimates [15]. For incidence calculations by race and ethnicity, multiple imputation was first conducted to fill in missing data on race and ethnicity using fully conditional specification. Ten imputations were performed using the following variables to inform imputation:

- Patient age, state and county of residence, outcome, sex, disease syndrome, race (to impute ethnicity) and ethnicity (to impute race)
- Calendar year of reported case
- County-level proportions of persons reported as White, Black or African American, Asian or Pacific Islander, American Indian or Alaska Native, Hispanic, or Non-Hispanic in U.S. Census data

Incidence and risk ratios by race and ethnicity were computed using Poisson regression on the imputed data. Incidence rates are reported as cases per 100,000 population.

Case fatality ratios (CFRs) were calculated using cases with known outcome as the denominator. Bivariate and multivariable analyses of CFR were conducted using logistic regression to examine patient and organism characteristics associated with CFR. Multivariable models included all variables that were significant at p < .05 in bivariate analysis. For analysis of CC, the five most frequently identified CCs were analyzed separately; all others were combined and classified as “other”.

This activity was reviewed by CDC, deemed not research, and was conducted consistent with applicable federal law and CDC policy.§.

## Results

### Improvements in surveillance

**Fig 1:**
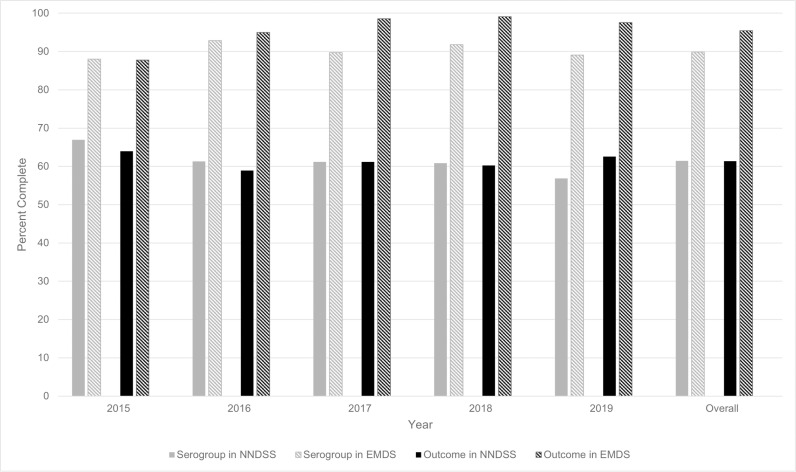
Completeness of Serogroup and Outcome in the National Notifiable Diseases Surveillance System and Enhanced Meningococcal Disease Surveillance over time, 2015–2019. Abbreviations: NNDSS, National Notifiable Diseases Surveillance System; EMDS, Enhanced Meningococcal Disease Surveillance.

Since EMDS began in 2015, notable improvements have been made to completeness of key variables over time. For key variables already collected as part of NNDSS, EMDS improved completeness over NNDSS by 21%-39% each year. Overall, for serogroup during 2015–2019, EMDS improved completeness over NNDSS from 61% to 90% complete. For patient outcome, EMDS improved completeness from 61% to 95% complete (Fig 1). Serogroup and outcome were significantly more complete in EMDS compared to NNDSS in all 5 years (p < .0001 for both variables each year).

**Fig 2:**
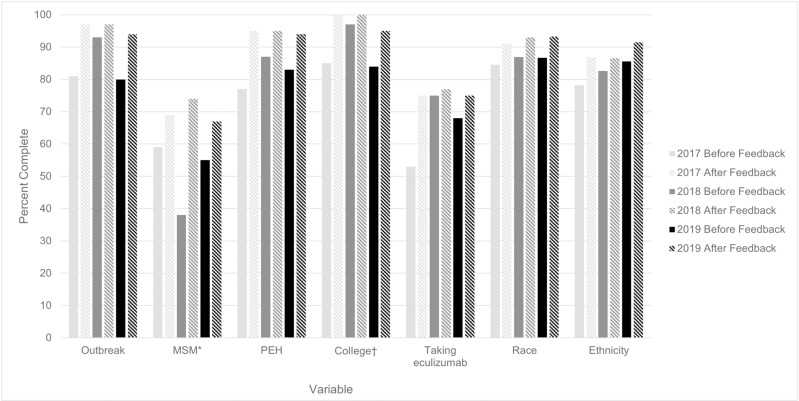
Completeness of Enhanced Meningococcal Disease Surveillance variables before and after feedback on missing data, 2017–2019 Abbreviation: MSM, men who have sex with men; PEH, person experiencing homelessness. *The percent complete for cases among MSM was calculated among cases in males aged 16 years and older. ^†^The percent complete for cases among college students was calculated among cases in people aged 18-24 years.

**Fig 3:**
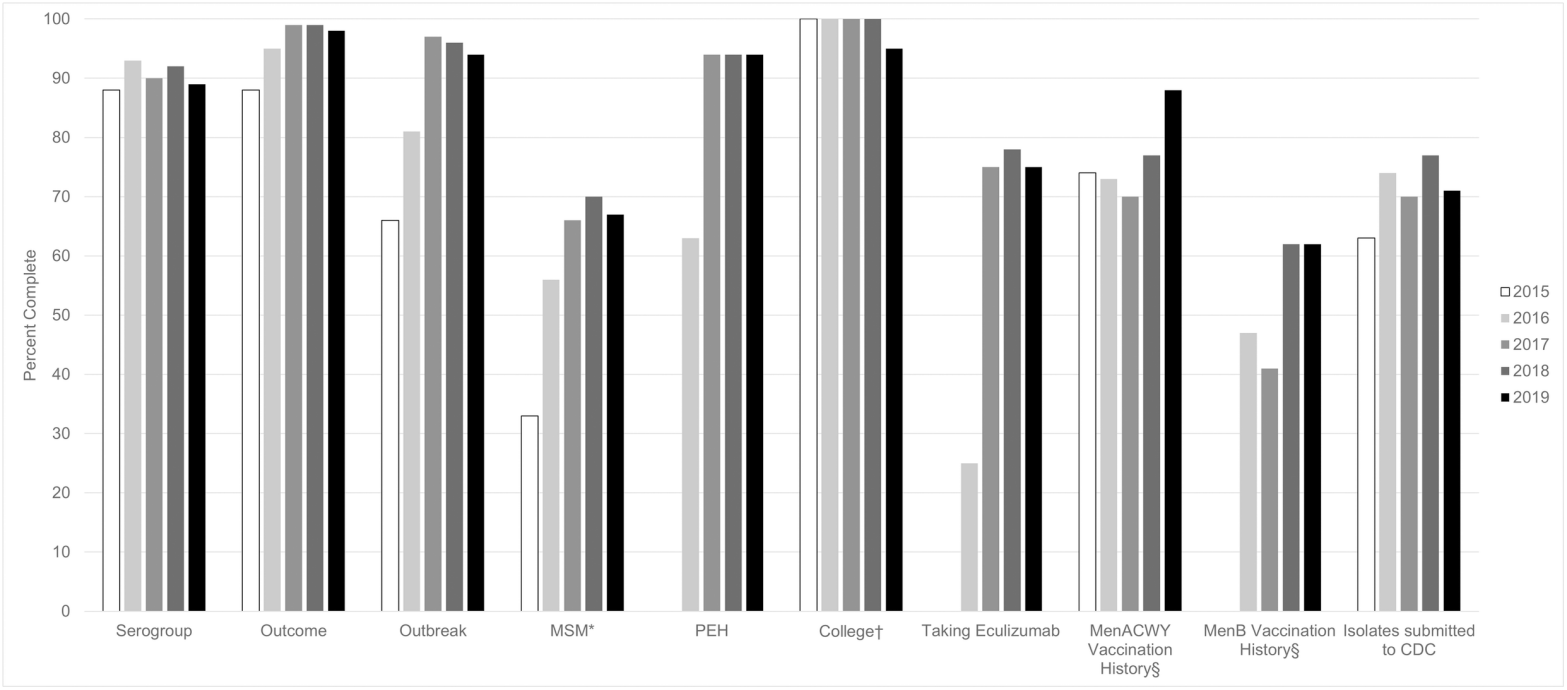
Completeness of Enhanced Meningococcal Disease Surveillance variables over time, 2015–2019. Abbreviation: MSM, men who have sex with men; PEH, person experiencing homelessness; MenACWY, quadrivalent meningococcal conjugate; MenB, serogroup B meningococcal. *The percent complete for cases among MSM was calculated among cases in males aged 16 years and older. ^†^The percent complete for cases among college students was calculated among cases in people aged 18-24 years. ^§^The percent complete for MenACWY and MenB vaccination history was calculated among cases in people aged 16-24 years.

In addition, supplemental variables were collected through EMDS to provide information on current meningococcal disease risk factors. Completeness was improved through feedback on missing data that began in 2017, with improvements of up to 36 percentage points comparing final data for each year to data available before the feedback was sent (Fig 2). Based on final data for each year, completeness of key variables improved an average of 18% from the first year the variable was collected to 2019 (Fig 3). The largest improvements were made for information on if the case was in a MSM or PEH and complement inhibitor use.

The feedback on missing data also improved completeness of race over NNDSS by 6–7% each year during 2017–2019 (Fig 2). Completeness of ethnicity improved over NNDSS by 4–9% each year during 2017–2019.

Improvements have also been made to the percent of cases with isolates submitted to CDC for molecular typing. The percent of cases with available isolates at CDC improved from 63% in 2015 to 71% in 2019 (Fig 3), with the highest percent in 2018 (77%). Overall, isolates were available at CDC for 71% of cases.

Final completeness of key variables was 90.9% for race, 87.0% for ethnicity, 86.7% for outbreak association, 99.2% for college attendance among people aged 18–24 years, 58.2% for information on history of sex with men among men aged ≥16 years, 72.5% for information on housing insecurity, 75.5% for MenACWY vaccine history among people aged 16–24 years and 50.8% for MenB vaccine among people aged 16–24 years.

### Meningococcal disease epidemiology: 2015–2019

**Fig 4:**
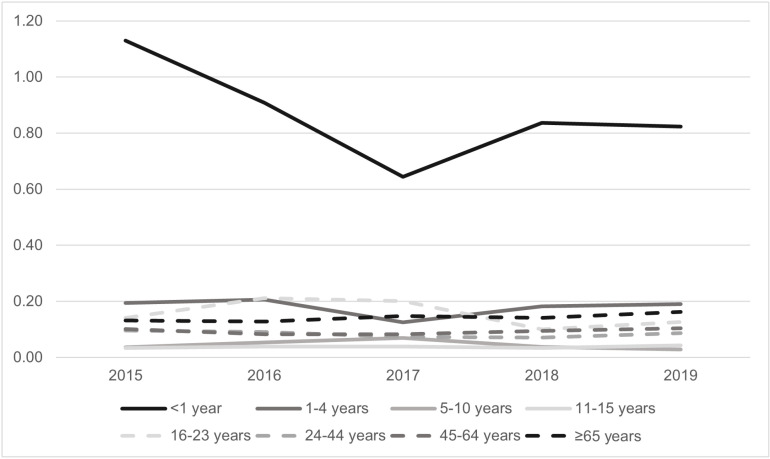
Incidence of Meningococcal Disease by Age Group, United States, 2015–2019.

**Fig 5:**
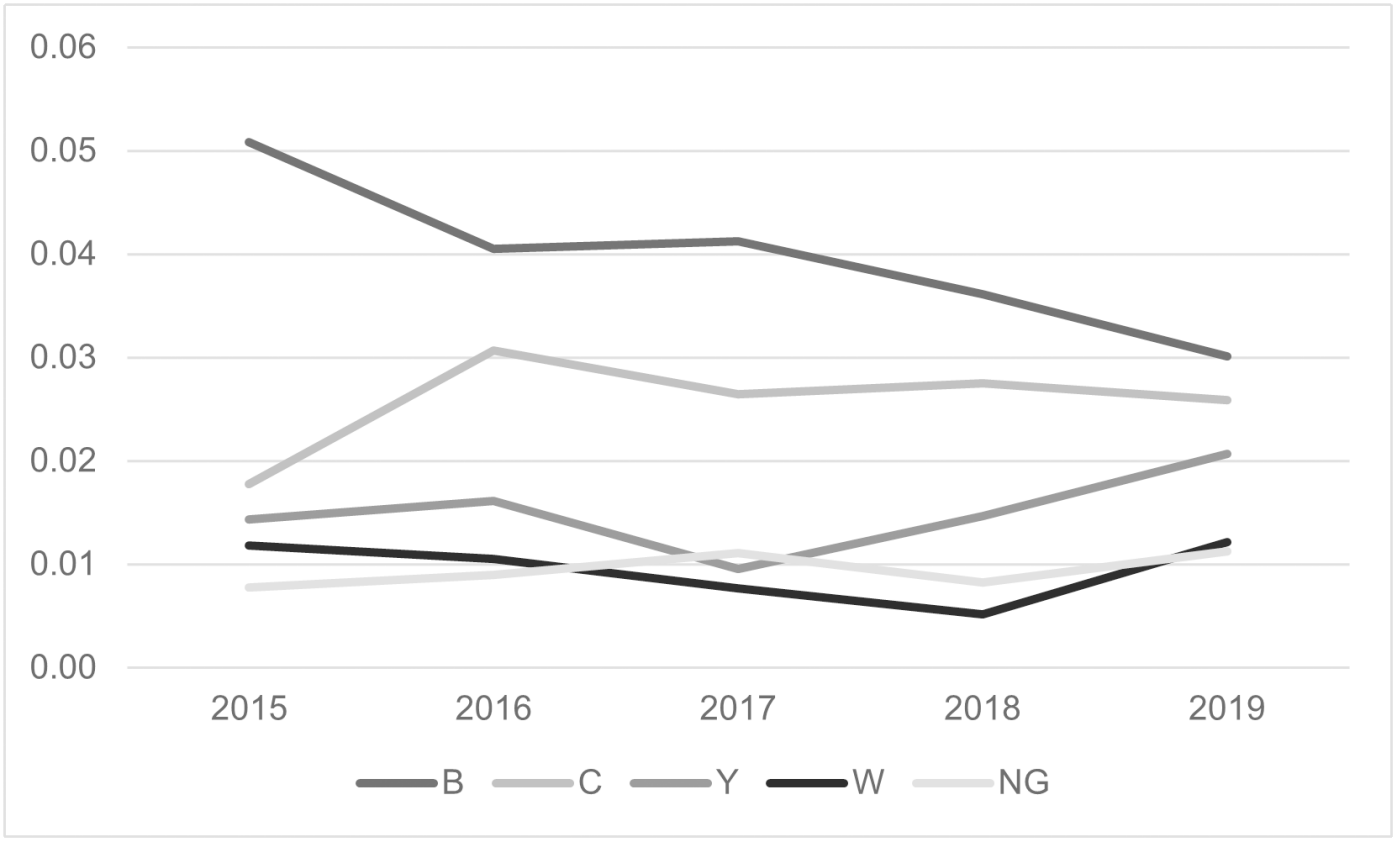
Incidence of Meningococcal Disease by Serogroup, United States, 2015–2019.

A total of 1,806 confirmed and probable meningococcal disease cases were reported through NNDSS from 2015–2019. The average annual incidence was 0.11 cases per 100,000 population. Average annual incidence ranged from 0.03 to 0.50 cases per 100,000 population by state. Incidence also varied by age group and serogroup; it was highest among children aged <1 year (0.87 cases per 100,000 population) (Fig 4) and for serogroup B (0.04 cases per 100,000) (Fig 5).

Of the 1,806 cases, 1,782 (98.7%) were confirmed cases according to the CSTE case definition and 24 (1.3%) were probable cases. Of the confirmed cases with known lab confirmation method, 1,437/1,563 (91.9%) were confirmed by culture, 126 (8.1%) were confirmed by PCR.

### Race and ethnicity

Of patients with known race, 1,195/1,642 (72.8%) cases occurred among White patients, 285 (17.4%) among Black patients, and 162 (9.9%) among patients of other racial groups (Table 1). The proportions were the same after imputation. After imputation, the average annual incidence was 0.10 (95% confidence interval [CI]: 0.10–0.11) cases per 100,000 population among White persons, 0.14 (95% CI: 0.12–0.15) per 100,000 population among Black or African American persons, 0.10 (95% CI: 0.06–0.14) per 100,000 population among American Indian or Alaska Native persons, and 0.06 (95% CI: 0.05–0.08) per 100,000 population among Asian or Pacific Islander persons.

**Table 1:** Characteristics of Meningococcal Disease Cases by Serogroup, United States, 2015–2019.

Characteristic	Serogroup B (n = 645)	Serogroup C (n = 417)	Serogroup W (n = 154)	Serogroup Y (n = 245)	Nongroupable (n = 154)	Other/ Unknown Serogroup^*^ (n = 191)	Total
	N (%^†^)	N (%^†^)	N (%^†^)	N (%^†^)	N (%^†^)	N (%^†^)	N (%^†^)
Age Group (years)							
<1	110 (17.1)	20 (4.8)	5 (3.3)	10 (4.1)	8 (5.2)	16 (8.4)	169 (9.4)
1–4	64 (9.9)	42 (10.1)	8 (5.2)	10 (4.1)	7 (4.6)	12 (6.3)	143 (7.9)
5–10	20 (3.1)	17 (4.1)	2 (1.3)	2 (0.8)	7 (4.6)	7 (3.7)	55 (3.1)
11–15	21 (3.3)	9 (2.2)	1 (0.7)	1 (0.4)	4 (2.6)	3 (1.8)	39 (2.2)
16–23	160 (24.8)	20 (4.8)	8 (5.2)	15 (6.1)	43 (27.9)	22 (11.5)	268 (14.8)
24–44	103 (16.0)	115 (27.6)	29 (18.8)	50 (20.4)	39 (25.3)	44 (23.0)	380 (21.0)
45–64	95 (14.7)	112 (26.9)	49 (31.8)	66 (26.9)	20 (13.0)	47 (24.6)	389 (21.5)
≥65	72 (11.2)	82 (19.7)	52 (33.8)	91 (37.1)	26 (16.9)	40 (20.9)	363 (20.1)
Sex							
Male	319 (49.5)	240 (57.6)	73 (47.4)	118 (48.2)	79 (51.3)	103 (53.9)	932 (51.6)
Female	326 (50.5)	177 (42.4)	81 (52.6)	127 (51.8)	75 (48.7)	88 (46.1)	874 (48.4)
Race							
White	476 (80.7)	264 (68.8)	103 (71.0)	147 (65.9)	81 (59. 6)	124 (75.6)	1195 (72.8)
Black or African American	58 (9.8)	71 (18.5)	32 (22.1)	48 (21.5)	46 (33.8)	30 (18.3)	285 (17.4)
Other	56 (9.5)	49 (12.8)	10 (6.9)	28 (12.6)	9 (6.6)	10 (6.1)	162 (9.9)
Ethnicity							
Hispanic or Latino	86 (15.6)	69 (18.2)	21 (14.9)	47 (21.6)	20 (16.1)	25 (16.0)	268 (17.1)
Non-Hispanic or non-Latino	466 (84.4)	311 (81.8)	120 (85.1)	171 (78.4)	104 (83.9)	131 (84.0)	1303 (82.9)
County of Residence							
Rural	103 (16.1)	38 (9.2)	23 (14.9)	29 (12.0)	13 (8.4)	48 (25.4)	254 (14.2)
Urban	536 (83.9)	376 (90.8)	131 (85.1)	213 (88.0)	141 (91.6)	141 (74.6)	1538 (85.8)
Outbreak Associated							
Yes	42 (7.4)	82 (22.3)	4 (2.9)	5 (2.3)	2 (1.4)	1 (0.8)	136 (8.7)
No	523 (92.6)	285 (77.7)	133 (97.1)	213 (97.7)	143 (98.6)	132 (99.2)	1429 (91.3)
Experiencing homelessness							
Yes	5 (1.1)	31 (8.9)	3 (2.7)	4 (2.2)	2 (1.7)	1 (1.0)	46 (3.5)
No	448 (98.9)	317 (91.1)	107 (97.3)	177 (97.8)	113 (98.3)	101 (99.0)	1263 (96.5)
College student (among 18–24 year olds)							
Yes	102 (71.3)	2 (8.7)	2 (22.2)	6 (37.5)	26 (60.5)	5 (25.0)	143 (56.3)
No	41 (28.7)	21 (91.3)	7 (77.8)	10 (62.5)	17 (39.5)	15 (75.0)	111 (47.3)
MSM (among men ≥ 16 years old)							
Yes	7 (6.8)	61 (43.3)	2 (7.4)	7 (10.5)	4 (10.5)	4 (11.4)	85 (20.7)
No	96 (93.2)	80 (56.7)	25 (92.6)	60 (89.6)	34 (89.5)	31 (88.6)	326 (79.3)

Abbreviation: MSM, men who have sex with men

* Includes 8 serogroup E cases

† Percent calculated of those with known response. Percent missing by variable: Race 9.0%, Ethnicity 13.0%, County of Residence 0.8%, Outbreak Associated 13.4%, Experiencing homelessness 27.5%, College student 0.8%, MSM 41.8%.

Of patients with known ethnicity, 268/1,571 (17.1%) cases occurred among Hispanic or Latino patients and 1,303 (82.9%) cases occurred among non-Hispanic or non-Latino patients (Table 1). Proportions were similar after imputation. The average annual incidence was 0.10 (95% CI: 0.09–0.12) cases per 100,000 population among Hispanic or Latino persons and 0.11 (95% CI: 0.11–0.12) among non-Hispanic or non-Latino persons.

### Risk factors and outbreak association

Of cases with known information, 136/1,565 (8.7%) were associated with an outbreak, 143/254 (56.3%) among people aged 18–24 years were in people attending college, 85/411 (20.7%) among men aged ≥16 years were identified as MSM, 46/1,309 (3.5%) were among PEH. Differences in meningococcal disease epidemiology were observed by serogroup, including almost all outbreak cases (124/136, 91.2%) being either serogroup B or C, most cases among college students were serogroup B (102/143, 71.3%), most cases among MSM and PEH were serogroup C (61/85, 71.8% and 31/46, 67.4% respectively), and a higher proportion of serogroup B cases were White while a higher proportion of nongroupable cases were Black or African American (Table 1).

### Case fatality

Among 1,724 cases with known outcome, the overall CFR for 2015–2019 was 12.8%. In bivariate analysis, CFR decreased each year, but year overall was not significantly associated with CFR. CFR did not differ significantly by sex, race, ethnicity, rural or urban county of residence, or association with an outbreak (Table 2). The CFR increased with increasing age and was significantly higher in cases among patients aged ≥65 years compared to cases among patients aged <1 year (OR 1.96 (95% CI: 1.12–3.41)). The CFR was higher for serogroup C and Y compared to B, but overall serogroup was not significantly associated with CFR. The CFR was significantly lower for CC103 compared to other CCs (OR 0.46 (95% CI: 0.23–0.91)). In multivariable analysis including age and CC, the only difference that remained significant was that CFR was significantly lower for CC103 compared to other CCs (OR 0.44 (95% CI: 0.22–0.89)).

**Table 2:** Meningococcal Disease Deaths and Case Fatality Ratio by Epidemiologic Characteristics, United States, 2015–2019.

		Bivariate Analysis		Multivariable Analysis	
	No. of deaths (CFR^*^)	Odds Ratio (95% CI)	P	Odds Ratio (95% CI)	P
N	221 (12.8)	–			
Year			.2		–
2015	52 (15.8)	Reference		–	
2016	49 (13.7)	0.85 (0.55-1.29)		–	
2017	45 (13.0)	0.80 (0.52-1.23)		–	
2018	40 (12.3)	0.75 (0.48-1.16)		–	
2019	35 (9.6)	**0.56 (0.36-0.89)**		–	
Sex			.8		–
Male	113 (12.6)	Reference		–	
Female	108 (13.1)	1.04 (0.79-1.38)		–	
Race			.1		–
White	142 (12.4)	Reference		–	
Black	44 (16.4)	1.38 (0.96-2.00)		–	
Other	16 (10.2)	0.80 (0.47-1.39)		–	
Ethnicity			.2		–
Hispanic or Latino	25 (9.8)	Reference		–	
Non-Hispanic or non-Latino	160 (12.7)	1.34 (0.86-2.08)		–	
County of Residence			.9		–
Rural	32 (13.1)	Reference		–	
Urban	188 (12.8)	0.97 (0.65-1.45)		–	
Outbreak Associated			.9		–
Yes	17 (12.5)	Reference		–	
No	172 (12.3)	0.98 (0.58-1.67)		–	
Age group			.0021		.1
<1 year	18 (11.0)	Reference		Reference	
1-4 years	16 (11.5)	1.05 (0.51-2.14)		0.81 (0.33-2.00)	
5-10 years	3 (5.9)	0.50 (0.14-1.78)		0.67 (0.18-2.53)	
11-15 years	4 (11.1)	1.01 (0.32-3.18)		1.29 (0.39-4.34)	
16-23 years	22 (8.4)	0.74 (0.38-1.42)		0.71 (0.33-1.54)	
24-44 years	39 (10.8)	0.98 (0.54-1.77)		0.80 (0.40-1.63)	
45-64 years	51 (14.0)	1.31 (0.74-2.32)		1.03 (0.52-2.05)	
≥65 years	68 (19.5)	**1.96 (1.12-3.41)**		1.55 (0.80-2.97)	
Serogroup			.1		–
B	61 (9.7)	Reference		–	
C	60 (14.9)	**1.62 (1.11-2.38)**		–	
NG	21 (13.8)	1.49 (0.88-2.53)		–	
W	20 (13.9)	1.50 (0.87-2.57)		–	
Y	37 (15.6)	**1.72 (1.11-2.66)**		–	
Clonal Complex			.0478		.1
Other CC	45 (13.6)	Reference		Reference	
CC11	46 (16.6)	1.27 (0.81-1.98)		1.29 (0.82-2.04)	
CC23	21 (15.0)	1.12 (0.64-1.96)		1.01 (0.57-1.79)	
CC32	15 (10.0)	0.71 (0.38-1.31)		0.81 (0.43-1.52)	
CC41/44	19 (10.8)	0.77 (0.44-1.36)		0.78 (0.44-1.39)	
CC103	11 (6.7)	**0.46 (0.23-0.91)**		**0.44 (0.22-0.89)**	

Abbreviations: CFR, case fatality ratio; CI, confidence interval; CC, clonal complex

* Case fatality ratio (CFR): deaths per 100 cases with known outcome.

### Vaccination history

The proportion of patients aged 16–24 years that received MenACWY vaccine increased over time from 65% in 2015 to 84% in 2019. Of the patients that received MenACWY vaccine, only 16/176 (9%) had disease caused by serogroups A, C, W, or Y. MenB vaccine history was complete for 130 (44%) patients aged 16–24 years. The proportion of patients that received one or more doses of MenB vaccine increased over time from 0% in 2015 to 29% in 2019. Of the patients that received MenB vaccine, 6/13 had disease caused by serogroup B. Five of the 6 were previously described [16] and the sixth only received 1 dose of MenB-4C 17 months before onset.

### Laboratory results

Serogroups B (29.6–49.4% of cases) and C (17.3–29.8% of cases) were predominant in all five years. 1,267 isolates at CDC had CC results available. For serogroup B, CC32 was the most common CC in 2015–2017, but CC41/44 became predominant in 2018 and 2019 (Table 3). For serogroup C, CC11 was most common in 2015–2017, but CC103 became predominant in 2018 and 2019, accounting for more than 60% of serogroup C cases in 2019. Of the 159 serogroup C CC103 cases, 157 (98.7%) were sequence type 2006, and many of these cases nationwide were genomically closely related. Of cases with known information, a smaller proportion of serogroup C CC103 cases were associated with outbreaks (21/132, 15.9%) compared to other serogroup C cases (61/235, 26.0%) and a larger proportion were in PEH (22/140, 15.7% vs. 9/208, 4.3% respectively). The age and race/ethnicity of serogroup C CC103 cases was similar to other serogroup C cases.

**Table 3:** Clonal Complex of Meningococcal Disease Isolates by Serogroup and Year, United States, 2015–2019.

	2015 (n = 235)	2016 (n = 278)	2017 (n = 237)	2018 (n = 252)	2019 (n = 265)
CC^*^	N (%^†^)	N (%^†^)	N (%^†^)	N (%^†^)	N (%^†^)
Serogroup B					
CC41/44	32 (28.1)	31 (29.8)	26 (29.9)	43 (44.8)	23 (33.3)
CC32	42 (36.8)	34 (32.7)	27 (31.0)	17 (17.7)	16 (23.2)
CC213	11 (9.7)	5 (4.8)	2 (2.3)	3 (3.1)	6 (8.7)
Other	29 (25.4)	34 (32.7)	32 (36.8)	33 (34.4)	24 (34.8)
Serogroup C					
CC103	19 (40.4)	32 (38.6)	28 (36.8)	38 (46.3)	42 (60.9)
CC11	26 (55.3)	44 (53.0)	35 (46.1)	30 (36.6)	16 (23.2)
CC32	0	1 (1.2)	7 (9.2)	1 (1.2)	0
Other	2 (4.3)	6 (7.2)	6 (7.9)	13 (15.9)	11 (15.9)
Nongroupable (NG)					
CC41/44	3 (18.8)	5 (22.7)	4 (16.0)	2 (9.5)	3 (9.1)
CC1157	2 (12.5)	4 (18.2)	3 (12.0)	2 (9.5)	6 (18.2)
CC11	2 (12.5)	2 (9.1)	2 (8.0)	3 (14.3)	3 (9.1)
Other	9 (56.3)	11 (50.0)	16 (64.0)	14 (66.7)	21 (63.6)
Serogroup W					
CC11	16 (61.5)	23 (82.1)	19 (86.4)	10 (66.7)	32 (86.5)
CC22	10 (38.5)	4 (14.3)	3 (13.6)	4 (26.7)	5 (13.5)
CC167	0	1 (3.6)	0	0	0
Other	0	0	0	1 (6.7)	0
Serogroup Y					
CC23	23 (71.9)	29 (70.7)	18 (66.7)	23 (60.5)	39 (68.4)
CC167	8 (25.0)	3 (7.3)	3 (11.1)	7 (18.4)	9 (15.8)
CC174	1 (3.1)	6 (14.6)	4 (14.8)	2 (5.3)	5 (8.8)
Other	0	3 (7.3)	2 (7.4)	6 (15.8)	4 (7.0)

Abbreviations: CC, clonal complex

* Data presented for the 3 most common clonal complexes in each serogroup during 2015–2019.

† Percent calculated of those with known clonal complex. Clonal complex was known for 71% of cases during this time period.

## Discussion

EMDS enhanced the availability of data compared to NNDSS alone, improving completeness of previously collected variables and adding the ability to collect additional variables relevant to the current epidemiology of meningococcal disease and isolates from cases. Using the improved data collected through EMDS, we found that incidence of meningococcal disease in the U.S. from 2015–2019 was lower than previous years, but differences in incidence by age group and race remained consistent with previous years, with incidence being highest among children aged <1 year and among Black or African American persons [1]. Consistent with previous years, serogroups B and C were the most common causes of disease [1]. Whole genome sequencing at CDC of isolates submitted as part of EMDS revealed expansion of CC41/44 (serogroup B) and CC103 (serogroup C) in the U.S. during the first five years of enhanced surveillance.

Completeness of key variables in EMDS improved over time by an average of 18% from the first year the variable was collected to 2019. Our data show that in addition to improvements over time, the feedback on missing data before finalizing data each year – initiated in 2017 – led to large improvements to completeness of key variables. While notable improvements were made to completeness of key variables from 2015 to 2019, completeness declined in 2019 compared to 2018. This is likely because 2019 data were closed out during 2020 when jurisdictions were dealing with competing priorities related to the COVID-19 pandemic. It will be important to ensure completeness does not continue to decline.

The more complete outcome data available through EMDS allowed for an analysis of factors associated with CFR. The overall CFR was lower than previously reported [1] and decreased from 2015–2019, but differences were observed by age group and CC. The CFR observed in the U.S. is higher than reported in many other developed countries [17], and this may be due in part to differences observed in serogroup and CC in different countries. It will be important to continue monitoring changes in circulating CCs and serogroups, along with patient age, to better understand variations in CFR over time. Additionally, data regarding time from disease onset to hospitalization were not available; further investigation in this area may help better illuminate the impact of time to seek care on CFR.

A key element of EMDS is collection of meningococcal disease vaccination history of patients, which helps monitor the impact of existing vaccine recommendations. While vaccination history completeness among 16–24 year olds ranged from 41% to 88% complete for MenB and MenACWY vaccine from 2015–2019, the information collected still sheds light on how vaccine coverage is changing among meningococcal disease cases over time and allows us to monitor meningococcal disease cases in previously vaccinated persons [16,18]. Additional data from vaccine effectiveness studies would be helpful in conjunction with these data to better understand the impact of existing vaccine recommendations in specific groups.

Isolate submission as part of EMDS is also critical to monitor meningococcal disease epidemiology. In addition to understanding changes in circulating serogroups CCs over time, isolate submission allows for monitoring of antimicrobial resistance as previously reported [19] and monitoring predicted strain coverage for MenB vaccines. Since EMDS began in 2015, the percent of cases with isolates available at CDC increased, but in 2019, 29% of cases still did not have an isolate available at CDC for additional testing. Additional communications on the importance of isolates to jurisdiction health departments may help improve submission of meningococcal disease isolates to CDC.

In addition to improving completeness of key variables and submission of isolates, EMDS provides flexibility to add new variables as they become epidemiologically relevant. In 2019, EMDS began collecting more detailed risk factor information for cases in college students, including year in school, residence type, and Greek life participation in response to university outbreaks of meningococcal disease. This flexibility allows EMDS to provide the most relevant data to understand current epidemiology.

Despite the improvements EMDS has made to meningococcal disease surveillance data completeness and quality, limitations to the data still exist. There are persistent gaps in completeness for some key variables, which makes it likely that some patients are being misclassified, e.g., as non-MSM due to missing data on MSM status. In addition, EMDS focuses on a limited number of high priority variables to improve data completeness, but this means there may be additional variables of interest that are not currently collected.

EMDS is an important part of national meningococcal disease surveillance as it allows collection of key variables that are not collected through NNDSS and facilitates improvements in variable completeness for both NNDSS and supplemental variables. Procedures implemented in EMDS to improve data quality can be a model for other surveillance systems. High quality data collected through EMDS supplement data collected through NNDSS and are critical to better understand risk factors for meningococcal disease and inform policy for prevention and control of meningococcal disease.

^§^ See, e.g., 45 C.F.R. part 46.102(l)(2), 21 C.F.R. part 56; 42 U.S.C. §241(d); 5 U.S.C. §552a; 44 U.S.C. §3501 et seq.
